# Anti-inflammatory potential of Quercetin in COVID-19 treatment

**DOI:** 10.1186/s12950-021-00268-6

**Published:** 2021-01-28

**Authors:** Ali Saeedi-Boroujeni, Mohammad-Reza Mahmoudian-Sani

**Affiliations:** 1 Department of Immunology, Faculty of Medicine, Ahvaz Jundishapur University of Medical, Sciences, Ahvaz, Iran; 2 Abadan School of Medical Sciences, Abadan, Iran; 3 ImmunologyToday, Universal Scientific Education and Research Network (USERN), Tehran, Iran; 4 Thalassemia and Hemoglobinopathy Research Center, Health Research Institute, Ahvaz Jundishapur University of Medical Sciences, Ahvaz, Iran

**Keywords:** COVID-19, SARS-CoV-2, Quercetin, NLRP3 inflammasome, TXNIP, SIRT1

## Abstract

SARS-CoV-2 is a betacoronavirus causing severe inflammatory pneumonia, so that excessive inflammation is considered a risk factor for the disease. According to reports, cytokine storm is strongly responsible for death in such patients. Some of the consequences of severe inflammation and cytokine storms include acute respiratory distress syndrome, acute lung injury, and multiple organ dysfunction syndromes. Phylogenetic findings show more similarity of the SARS-CoV-2 virus with bat coronaviruses, and less with SARS-CoV. Quercetin is a carbohydrate-free flavonoid that is the most abundant flavonoid in vegetables and fruits and has been the most studied to determine the biological effects of flavonoids. Inflammasomes are cytosolic multi-protein complexes assembling in response to cytosolic PAMP and DAMPs, whose function is to generate active forms of cytokines IL-1β and IL-18. Activation or inhibition of the NLRP3 inflammasome is affected by regulators such as TXNIP, SIRT1 and NRF2. Quercetin suppresses the NLRP3 inflammasome by affecting these regulators. Quercetin, as an anti-inflammatory, antioxidant, analgesic and inflammatory compound, is probably a potential treatment for severe inflammation and one of the main life-threatening conditions in patients with COVID-19.

## Introduction

Acute respiratory infection was reported in several patients in Wuhan, Hubei Province, China in December 2019. Due to the direct or indirect connection of many people with the Huanan Seafood Wholesale Market in Wuhan, this place is known to be the source of the new coronavirus outbreak that has affected many people globally now [1]. Investigations documented that the novel coronavirus has genetically close similarity with the SARS virus, which spread to 29 countries from China in 2002 and caused the death of 744 people. Based on the findings of the gene sequencing and the similarity of spike proteins on the virus surface, similar to the SARS virus, the novel coronavirus probably uses angiotensin-converting enzyme 2 receptors (ACE2) to penetrate the cell. Phylogenetic findings show more similarity of the SARS-CoV-2 virus with bat coronaviruses [2, 3], and less with SARS-CoV. Based on the sequencing of viruses isolated from five patients, the SARS-CoV-2 genome sequence was 96% similar to bat coronavirus [1, 4]. The coronavirus genome consists of a 30-kb single-stranded RNA (+ssRNA) with a 5p cap and a poly-A tail. The proofreading ability of coronaviruses distinguishes them from other RNA viruses, which is due to the presence of 3′-5 ‘exoribonucleases. The SARS-CoV-2 is a betacoronavirus that causes severe respiratory syndrome by involving the lower respiratory tract. The infectivity rate of SARS-CoV-2 is much higher than SARS-CoV [5]. High levels of infectivity and mortality in ICU patients with severe disease are two challenging issues. Most Chinese patients deceased from COVID-19 were over 60 years of age and associated with underlying diseases, such as abdominal tumors, chronic liver disease, myocarditis, and kidney failure and cardiovascular disease [6, 7]. In this study, while reviewing the mechanisms of action of Quercetin, especially with the focus on the effect of the drug on immunological responses, we intend to discussed the effectiveness of this drug in patients with COVID-19.

### A body of literature

#### NLRP3 inflammasome

Pattern Recognition Receptors (PRRs) are among the key components of the innate immune system. Various types of PRRs have been identified so far, including the cytoplasmic nod-like receptor (NLR) protein family that develops the inflammation through the identification of pathogen-associated molecular patterns)PAMP(and Damage-associated molecular patterns)DAMP(, as well as the recruitment of other proteins involved in the signaling pathway. The NLR family differs in executive domain. The inflammasomes are cytosolic multi-protein complexes assembling in response to cytosolic PAMPs and DAMPs, whose function is to generate active forms of cytokines interleukin 1 beta (IL-1β) and IL-18. These two cytokines are initially inactive and then, under the influence of caspase 1, are converted to their active form by the proteolytic method, creating an inflammatory response. The NLR family sensors in different inflammasome complexes are NLRB and NLRC4, and at least six receptors exist within the NLRP subfamily. Types of cytosolic sensors out of the NLRP family include Absent in melanoma 2 (AIM2) and IFI16, which contain a DNA sensor domain and an N-terminal pyrin domain that can form inflammasome. NLR family pyrin domain containing 3 (NLRP3) inflammasome has been studied more than any other type of inflammasomes and has an important role in anti-inflammatory and antiviral responses. The activation of NLRP3 inflammasome induces the production of reactive oxygen species (ROS) that are sent into the cytosol through damage to the lysosomal membrane. The activated NLRP3 inflammasome also causes a type of cell death associated with inflammation in macrophages and dendritic cells, called pyroptosis. In this pathway, a protein called gasdermin D is cleaved by caspase 1, and subsequently the formation of membrane pores in the cell. The activated NLRP3 inflammasome exacerbates inflammation to microbial clearance and leads to septic shock [8, 9].

### Flavonoids and quercetin as anti-inflammatory and NLRP3 inflammasome inhibitors

Flavonoids, or phenolic acids, belong to the group of water-soluble plant pigments, which are responsible for the antioxidant capacity of fruits and plants. Epidemiological evidence confirms the reduced risk of cardiovascular disease and brain disease by eating lots of vegetables and fruits (flavonoids). Flavonoids are the most abundant polyphenols in the human diet. Flavonoids include anthocyanins, flavonols, flavanones, flavones, and isoflavones and quercetin (3,3′,4′,5,7-pentahydroxyflavone) is one of the most important compounds in the group of flavonoids. Recent research has shown that flavonoids are absorbed in humans and excreted unchanged or flavonoid metabolites through urine and feces. More than 4000 different flavonoids have been identified in the main groups of flavonoids [10]. Quercetin is a carbohydrate-free flavonoid that is the most abundant flavonoid in vegetables and fruits and has been the most widely studied to determine the biological effects of flavonoids [11]. The compound has a wide range of health, biological, antioxidant, anti-inflammatory, immune system regulation, and cardioprotective and neuroprotective effects. The neuroprotective effects of quercetin are mainly due to its antioxidant capacity and free radical scavenging ability. Quercetin supplementation can affect mitochondrial biogenesis, energy production and electron transport chain performance, ROS production modification, and mitochondrial defect modification. It can also pass through the blood brain barrier [12].

### Importance of NLRP3 inflammasome inhibition in the treatment of inflammatory diseases

The NLRP3 inflammasome is one of the most important research areas in immunology. Our understanding of the molecular mechanisms of NLRP3 inflammasome activation can be helpful in the treatment of inflammatory diseases. A number of NLRP3 inflammasome inhibitors have been reported to date, including those that inhibit directly NLRP3 or indirectly the components of the NLRP3 inflammasome or related signaling pathways [13]. The IL-1β signaling inhibition has been successful in the treatment of inflammatory disorders associated with NLRP3 inflammasome. Canakinumab as an IL-1β neutralizing antibody, Anakinra as a recombinant human IL-1 receptor antagonist capable of inhibiting the binding of IL-1β and IL-1α, and rilonacept as IL-1 decoy receptor binding to IL-1β and IL-1α have been approved by the Food and Drug Administration (FDA) in the management of different inflammatory disorders [14]. However, IL-1β is only one of the proinflammatory cytokines involved in immunopathogenesis of diseases, which is itself the result of the activation of inflammation. Directly targeting NLRP3 inflammasome is a much less invasive and more cost-effective method, and more specific than blocking cytokines. The MCC950, initially known as the CRID3/CP-456773, is the strongest and most specific NLRP3 inflammasome inhibitor [15]. In vitro studies have shown that MCC950 can inhibit both focal and non-focal pathways of NLRP3 inflammasome activation in mice and human macrophages specifically without impairing the NLRP1, NLRC4 AIM2 inflammasome complexes, or without disrupting signaling pathways associated with Toll-like receptors (TLRs). There have been reports of the effectiveness of MCC950 in the treatment of a variety of immunopathological models, including CAPS, experimental autoimmune encephalomyelitis [16], Alzheimer’s disease [17], atherosclerosis [18], and cardiac arrhythmia [19]. A phase-II clinical trial that used MCC950 to treat rheumatoid arthritis was discontinued due to hepatotoxic effects. However, other studies have reported convincing reasons that have led future efforts to target NLRP3 inflammasome for the treatment of inflammatory diseases [20]. Drugs that have previously been approved by the US Food and Drug Administration or other human-used compounds that can effectively inhibit NLRP3 inflammasome could be a potential treatment in severe COVID-19 cases.

### TXNIP as a bridge between oxidative stress and NLRP3 inflammasome

Thioredoxin (Trx) molecule along with Thioredoxin reductase (TrxR) and NADPH are components of the Trx/TrxR complex, which plays a key role in many intracellular activities as one of the main regulators of the Redox system. Maintaining oxidant/antioxidant balance, phenotypic, functional and apoptotic homeostasis of immune cells and cancer cells are among the activities affected by this intracellular Trx/TrxR complex [21]. The thioredoxin interacting protein (TXNIP) gene is located on chromosome 1q21-1q23 in the same locus as T2DM. TXNIP is a central mediator that is associated with NLRP3 inflammasome activation by binding to NLRP3 as ROS-dependent manner after separation from Trx. Inflammatory activators such as uric acid crystals cause the TXNIP to be separated from Trx by the ROS-sensitive method, thus facilitating the binding of TXNIP to NLRP3 [22]. TXNIP is a major mediator of pancreatic β-cell dysfunction, one of the most important genes regulated in response to hyperglycemia. It inhibits glucose uptake and promotes the cleavage of caspase-1, which leads to glucose-dependent β-cell death [23].

### Quercetin inhibits NLRP3 inflammasome by affecting TXNIP

Fructose-incubated U937 and THP-1 cells have been shown to increase ROS production and expression of IL-1β, IL-18, and caspase-1 genes and proteins, all of which are evidence of NLRP3 inflammasome activity in these cells. Interestingly, the treatment of human and mouse macrophages with fructose increases the rate of NLRP3 exposure to TXNIP in the cytosol and thus activates NLRP3 inflammasome. The results of this study showed that quercetin and ascorbic acid owing to their antioxidant properties can prevent the activation of NLRP3 inflammasome through TXNIP and thus prevent the exacerbation of inflammation [24]. Other studies have also reported that the quercetin prevents the NLRP3 inflammasome activation through TXNIP. For example, a study evaluated the molecular mechanism of quercetin in rat models of spinal cord injury (SCI). The results revealed a significant increase in NLRP3 inflammasome activity, a sharp increase in proinflammatory cytokine production and secretion, and a significant reduction in the rate of ROS production by the quercetin in the SCI rats. An increase in ROS concentration has been identified as one of the prominent NLRP3 inflammasome stimulants that occur through TXNIP. Previous studies have shown that the quercetin is also a TXNIP inhibitor. In addition, the quercetin, as expected, was also able to inhibit the proinflammatory cytokine production and the NLRP3 inflammasome activation in these rats. In addition, the quercetin improved histopathological conditions and motor status in the SCI rats. This study also confirmed the anti-inflammatory effects of quercetin besides its therapeutic potential in diseases with inflammatory background and NLRP3 inflammasome activity, which is a known factor in the immunopathogenesis of COVID-19 [25]. In a study, differentiated THP-1 cells were treated with the monosodium urate (MSU) crystals to activate NLRP3 inflammasome. Of the 56 flavonoids tested, only flavone, apigenin, kaempferol and quercetin were significantly reduced the IL-1β production [26]. Diabetic nephropathy (DN) is one of the most debilitating consequences for people with diabetes. Overactivation of NLRP3 inflammasome due to overproduction of ROS and high glucose levels is one of the important factors in DN immunopathogenesis. A study of DN rats found that Dihydroquercetin (DHQ) besides other effects was able to effectively prevent the overproduction of ROS in the rat kidney cells and ultimately the activation of NLRP3 inflammasome. By inhibiting destructive inflammation, the DHQ thus prevented severe renal cell damage and eventually the risk of DN. This study showed well how quercetin is able to act as an effective anti-inflammatory factor by inhibiting NLRP3 inflammasome [27]. In a study, researchers investigated the possible role of TXNIP in type 1 diabetes-associated non-alcoholic fatty liver disease (NFALD) in the rat BRL-3A and human HepG2 cells. The results showed that the activation of NLRP3 inflammasome due to increased ROS production and under the influence of TXNIP factor plays a major role in the incidence of the disease. Severe inflammation plays a key role in the immunopathogenesis of these diseases. Quercetin and allopurinol significantly increased TXNIP expression, as well as inhibited NLRP3 inflammasome activation, increased SREBP-1c and SREBP-2, and synthesized fatty acids, increased ROS and IL-1β secretion in diabetic rat liver. Finally, the findings of this study showed that targeting liver TXNIP by quercetin and allopurinol may be a therapeutic target in the prevention of type 1 diabetes-associated NAFLD [28]. The endoplasmic reticulum (ER) stress-associated TXNIP/NLRP3 inflammasome activation is key factors in the immunopathogenesis of endothelial cell dysfunction. This signaling pathway eventually exacerbates inflammation, increases IL-1β production and kills endothelial cells. Quercetin, Luteolin and Epigallocatechin gallate (EGCG) reduce ROS production and inhibit TXNIP-NLRP3 inflammasome in endothelial cells and reduce their IL-1β production. In addition, these agents protect endothelial cells from death by apoptosis through the restoration of mitochondrial membrane potential (Deltapsim) and the inhibition of caspase-3 activity [29]. The results of this study clearly show how quercetin, while acting as an antioxidant and anti-inflammatory factor, can prevent the cells from apoptosis by inhibiting the apoptotic process. Evidence from patients with the severe form of COVID-19 shows high cellular degeneration besides severe inflammation, and these results clearly illustrate why quercetin can be a potential treatment for COVID-19. A study evaluated the effect of quercetin on the hypothalamus of high-fructose-fed rats. Findings from this in vivo study could provide evidence of quercetin-mediated downregulation of AMPK/TXNIP and subsequent inhibition of NF-κB /NLRP3 inflammasome pathway. Through this mechanism, the quercetin can reduce the hypothalamic inflammatory lesions [30].

### Effect of quercetin on SIRT1 as anti-inflammatory factor and NLRP3 inflammasome inhibitor

A recent study found that the quercetin improved diabetic encephalopathy in diabetic rats via the SIRT1/NLRP3 pathway. Interestingly, the quercetin was able to increase the expression of SIRT1 and decrease the expression of NLRP3 inflammasome components such as NLRP3 and adapter protein ASC and activated caspase-1. The quercetin also decreased the expression of proinflammatory cytokines, such as IL-1β and IL-18 [31]. The findings of this study are extremely important because SIRT1 is a pivotal factor. The SIRT1 is a class III histone deacetylases, involved in regulating various physiological processes, including cellular inflammation and metabolism. Emerging evidence has shown that the SIRT1 not only downregulates the NF-κB signaling pathway but also has anti-inflammatory functions in several tissues. The level of SIRT1 expression decreases with age. Studies have shown that the SIRT1 activation may be at least partially responsible for increasing life expectancy in mammals. Aging in humans is associated with chronic and mild inflammation called Inflammaging, predisposing many age-related chronic diseases [32]. The NF-κB is the main regulator in the development of the Inflammaging. The SIRT1 inhibits NF-κB gene expression through the deacetylation of RelA/p65 NF-κB subunit in lysine 310 [33]. A recent study reported that the SIRT1 is a potential therapeutic target for the prevention of pregnancy-related complications associated with inflammation [34]. Another study shows that the SIRT1 suppresses acute pulmonary inflammation during sepsis by controlling the NLRP3 inflammasome activation [35]. The NLRP3 inflammasome is involved in various central nervous system disorders by exacerbating the inflammation. In this study, it was found that the SIRT1 improved these disorders by inhibiting the NLRP3 inflammasome [36]. Another study found that the Doxofylline inhibited NLRP3 inflammasome via SIRT1 and thus prevented inflammation in lung epithelial cells [37]. Studies have shown that the GD15 is able to prevent acute pulmonary inflammation by increasing the expression of SIRT1 in the inflammation-caused lung injury of rats following the NLRP3 inflammasome activation [38]. The NLRP3 inflammasome is involved in the development of various Central nervous system (CNS) disorders by exacerbating the inflammation. The SIRT1 was able to help improve these disorders through the NLRP3 inflammasome inhibition [36].

### Effect of quercetin on **gut microbiome**

Gut microbiota has a profound effect on the homeostasis of local and systemic immune responses [39]. The association of dysbiosis with a range of diseases has been recognized, and inflammatory diseases are also affected by gut microbiome [40]. A study evaluated the effect of quercetin on gut microbiome balance and related gut-liver axis activation in rat models of NFALD and obesity. Metagenomic studies have shown the gut microbiome imbalance, called dysbiosis. Dysbiosis in NFALD includes an increase in Firmicutes/Bacteroidetes ratio as well as an increase in gram-negative *H. pylori* bacteria. Dysbiosis further exacerbates inflammation in a variety of ways. Activation of TLR4/NF-κB signaling pathway due to dysbiosis is associated with activation of NLRP3 inflammasome and ER stress. However, the quercetin restores the gut microbiome imbalance and leads to the cessation of the dysregulation of the expression of genes related to lipid metabolism by inhibiting the NLRP3 inflammasome activation caused by endotoxinemia [41]. This study is important because basically the inflammation caused by metabolism and the wrong lifestyle called metainflammation is a suitable platform for many inflammatory diseases, and it seems that the deadly reaction of the immune system of a group of people with COVID-19 is due to this type of inflammatory underlying causes.

### Quercetin and autophagy

Bacterial infections, such as *E. coli O157: H7* infection can cause severe inflammation and oxidant/antioxidant imbalance with NLRP3 inflammasome activation, high ROS production, mitochondrial membrane dysfunction and increased production of IL-1β and IL-18 cytokines. The findings showed that the quercetin could prevent NLRP3 inflammasome activation in *E. coli O157: H7* infection by maintaining the mitochondrial membrane integrity and inhibiting mitochondrial ROS release to cytosol. The *E. coli O157: H7* infection inhibits autophagy in the host cell. However, the quercetin by increasing the autophagy prevents overproduction of ROS and production of IL-1β and IL-18 [42]. Increased quercetin-mediated autophagy to prevent further inflammation is one of the most important findings of this study, which could be another reason for the positive effect of quercetin in the treatment of COVID-19. A study investigated the effect of extracts of water dropwort (EWD) and pharmacological molecules derived from hyperoside and isorhamnetin, which are derivatives of quercetin, on inflammatory responses especially NLRP3 inflammasome activation. The anti-NLRP3 inflammasome function of EWD, hyperoside and isorhamnetin has been well demonstrated in human and mouse macrophages. The EWD reduced the IL-1β secretion. In addition, it inhibited the formation of pyroptosome from NLRC4, NLRP3, and AIM2 inflammasomes without disturbing the expression of cytokines. The isorhamnetin selectively inhibited the activation of AIM2 and NLRP3 inflammasomes and the secretion of proinflammatory cytokine. The hyperoside selectively impaired the activation of AIM2 and NLRC4 inflammasomes, but not proinflammatory cytokine expression. In addition, all three EWD, isorhamnetin and hyperoside effectively inhibited caspase-1 activity. Therefore, hyperoside and isorhamnetin, as compounds derived from quercetin, can be considered as anti-inflammatory compounds, especially as NLRP3 inflammasome inhibitors [43]. A study evaluated the effect of three flavonoids of quercetin, naringenin and silymarin on the NLRP3 inflammasome activation. The results showed that quercetin was able to inhibit the activation of NLRP3 and AIM2 inflammasomes, but not NLRC4 inflammasome. The results of this study also showed that the effects of the above compounds occur in a completely independent way of autophagy. In addition, the quercetin inhibits the ASC oligomerization, which is essential for the NLRP3 inflammasome activation.

### Quercetin helps to establish TH17/Treg balance

Recent studies have shown the destructive role of TH17 lymphocytes and neutrophils as type 3 inflammation in many autoimmune disorders and inflammatory diseases. In fact, the imbalance between TH17 and its produced cytokines with Treg and its cytokines is a key phenomenon in the immunopathogenesis of many severe inflammatory diseases. Studies in animal models as well as patients with COVID-19 have shown severe neutrophil infiltration into lung tissue. In addition, various studies reported an increase in serum IL-17 in patients. The TH17/Treg imbalance can be considered a pathological event in COVID-19. In a study, collagen-induced arthritis (CIA) mice were treated with oral quercetin at a dose of 150 mg/kg, followed by the examination of clinical symptoms. Interestingly, the quercetin reduced TH17-related cytokines such as IL-17A and IL-21 and increased Treg-related cytokines such as IL-10 and TGF-β. Moreover, the quercetin reduced the percentage of Th17 cells and increased the percentage of Treg cells. In addition, a decrease in the NLRP3 inflammasome activation, IL-1β and caspase-1 was also clearly observed as a result of quercetin treatment. The quercetin also reduced inflammatory mediators including TNF-α, IL-1β, IL-6, PGE2, COX-2 and iNOS significantly. Importantly, all of the therapeutic effects mentioned for quercetin were lost as a result of the anti-HO1 siRNA. Therefore, it can be concluded that the quercetin is able to effectively improve CIA mice by establishing TH17/Treg balance, inhibiting NLRP3 inflammasome and suppressing inflammation through HO1 expression [44].

### Quercetin in metabolic syndrome

In another study, the fructose was used to induce hyperuricemia and dyslipidemia in rats, which activated NLRP3 inflammasome in kidney cells. Subsequently, the fructose-induced hyperuricemia and dyslipidemia were treated by allopurinol, quercetin and NLRP3 inflammasome inhibitors to improve signaling disturbances and reduce lipid accumulation. The results showed that a combination of the uric acid-lowering drug, allopurinol, and quercetin, as the NLRP3 inflammasome inhibitor, could play a key role in the immunopathogenesis in patients with severe inflammation caused by NLRP3 inflammasome activity [45].

### Effect of quercetin on the AIM2 inflammasome pathway

In a study, IFN-γ-primed keratinocytes were treated with double-stranded DNA viruses, called poly (dA: dT). In fact, this activates the AIM2 inflammasome. These cells were subsequently incubated by quercetin. The results of this study clearly showed that the quercetin could inhibit poly (dA: dT)-induced IL-18 production primarily by inhibiting the JAK2/STAT1 signaling pathway in IFN-γ-primed leukocytes. The inhibitory effect is due to the reduction of AIM2 and pro-caspase1 expression by the quercetin. This study actually shows another aspect of quercetin inhibitory potential in situations where severe inflammation is caused by viral invasion [46].

### Effect of quercetin on inflammatory macrophages

Many studies have shown that the quercetin acts as a protective factor in chronic cardiovascular disease (CVD) and related risk factors. Atherosclerosis is the leading cause of CVD that is reduced by using oral quercetin in animal models. The macrophages are one of the main innate immune cells involved in the development of atherosclerosis. The results of this study showed that the quercetin, like other similar substances, inhibited the ROS production and IL-6 secretion in oxLDL-stimulated macrophages. The quercetin also inhibited the production of IL-1β followed by NLRP3 inflammasome activation (under the influence of cholesterol crystals). apolipoprotein E-knockout mouse models of dyslipidemia intraperitoneally treated with quercetin showed smaller atheromatous lesions, decreased atherosclerosis, decreased of inflammatory lymphocyte and macrophage infiltration through coronary arteries [47]. It should be noted that severe inflammation, extensive infiltration of monocytes, lymphocytes and neutrophils, and overproduction of proinflammatory cytokine all play a key role in COVID-19 immunopathogenesis, especially in severe cases of acute lung injury (ALI) and acute respiratory distress syndrome (ARDS); accordingly, this study also shows the possible therapeutic effect of quercetin in COVID-19.

### Quercetin as an analgesic and anti-inflammatory

**Fig. 1:**
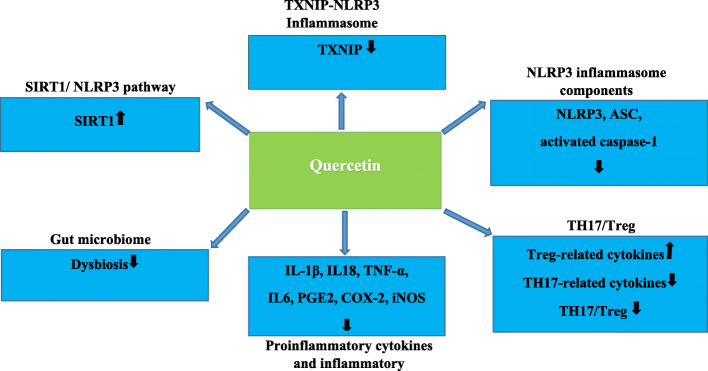
Molecular mechanisms of Quercetin action to manage COVID-19

A study evaluated the anti-inflammatory and analgesic effects of quercetin in MSU-induced gout. The study also compared the sensitivity of quercetin effects with naloxone, as a mu-opioid receptor antagonist. The results demonstrated that the quercetin inhibited MSU-induced mechanical hyperalgesia, leukocyte recruitment, production of TNFα and IL-1β, production of superoxide anion, NLRP3 inflammasome activation, NF-κB activation and expression of NLRP3 inflammasome complex mRNA. The pre-treatment of naloxone prevented all inhibitory effects of quercetin on MSU-induced gout. These results showed that the quercetin exerts its analgesic and anti-inflammatory effects on MSU-induced gout in a naloxone-sensitive manner [48]. Alcoholic hepatitis is characterized by inflammation and necrosis of the liver tissue due to excessive intake of alcohol and sometimes even causes liver failure. Oxidative stress and NLRP3 inflammasome activation play a key role in the immunopathogenesis of this destructive disease. The quercetin can increase Nrf2 expression and improve acute alcohol-induced liver injury in rats. The positive effects of quercetin in the treatment of alcoholic hepatitis are due to its antioxidant properties and inhibitory effect on the ROS/NF-κB/NLRP3 inflammasome /IL-1β/IL-18 pathways through the production of HO1. Meanwhile, the quercetin also increases the anti-inflammatory factor IL-10 independent of HO1. Thus, the quercetin increases IL-10 and HO-1 expression by inhibiting NLRP3 inflammasome activation and inflammatory factor secretion, and maintaining liver function in acute alcohol-induced injury [49]. The findings of this study raise hopes for a positive effect of quercetin to control the life-threatening uncontrolled inflammation in the patients with COVID-19 [49]. (Fig. 1) shows the most important Molecular Mechanisms of quercetin Action mentioned in this article.

Studies have shown that SARS-CoV-2 elevated inflammation and activates NLRP3 inflammasome, this leads to a cytokine storm and destructive inflammation and Causes ALI/ARDS in patients with COVID-19. Several important pathways, including those, TXNIP-NLRP3, NLRP3 inflammasome, SIRT1/ NLRP3 pathway, NLRP3 inflammasome components, TH17/Treg, proinflammatory cytokines and inflammatory mediators, have been identified as potential targets of Quercetin to manage COVID-19.

### Quercetin in COVID-19

A clinical trial on the effect of Quercetin on the prevention and treatment of COVID-19 has been registered in ClinicalTrials.gov with the ID code (NCT04377789). This clinical trial, which is being conducted in Turkey, aims to determine whether the daily use of two 500 mg tablets of Quercetin can prevent or treat the high-risk form of COVID-19. The results of this trial have not been published as of this writing. In Iran, we are conducting a clinical trial with the ID code (IRCT20200419047128N2) to determine the effect of Quercetin on the efficacy of antiviral drug regimen in COVID-19 patients. For this purpose, patients with the high-risk form of COVID-19 will be treated with Quercetin (two 500 mg tablets) and will undergo clinical examinations and also a study of inflammation-associated cytokines. In a recent study [43], researchers have assessed the ability of 100 secondary metabolites extracted from *Aframomum melegueta* to inhibit host furin. It has been shown that, like EBOLA and HIV, SARS-CoV-2 has a unique furin-like cleavage site (FCS), (RRAR), which makes it several times more contagious than other beta-coronaviruses such as SARS-CoV [50]. Also, the effect of the aforementioned compounds on SARS-CoV-2 targets such as 3C-like proteinase (Mpro/3CLpro), 2′-O-ribose methyltransferase (nsp16), and surface glycoprotein/ACE2 receptor interface has been investigated. Findings have shown that flavonoids such as Apigenin, Tectochrysin, and Quercetin have high binding power to SARS-CoV-2 targets. In a bioinformatics study, researchers have investigated the potential of compounds derived from Moringaoleifera in inhibiting SARS-CoV-2 proteases and made a comparison with known antiviral drugs. The results of this study have clearly demonstrated that much similar to well-known antiviral drugs like Lopinavir-Ritonavir, Maraviroc, Nelnavir, and Tipranavir, flavonoid compounds such as isoquercetin, quercetin, and dihydroquercetin can potentially exhibit antiviral properties through the inhibition of SARS-CoV-2 proteases like Mpro. In addition to this potential antiviral property, these compounds also have antioxidant properties that can help limit the damage of the severe form of COVID-19 [51].

## Conclusion

The SARS-CoV-2 pandemic has caused the death of over 1,030,028 people. Acute respiratory distress syndrome and acute lung injury resulting from cytokine storm and severe inflammation are considered as the leading cause of death in patients with severe COVID-19. Many clinical trials are currently underway to alleviate this destructive inflammation. The NLRP3 inflammasome inhibition, owing to its key role in inflammation, can be a more efficient, cost-effective and accurate method than merely inhibiting cytokines. The quercetin has an interesting inhibitory effect on inflammatory responses. On the other hand, it not only inhibits the production of NLRP3 inflammasome components and pro-IL-1β, but also suppresses inflammation through interference in various signaling pathways, especially NF-κB. The NLRP3 inflammasome is affected by regulators such as TXNIP, SIRT1 and NRF2, which play a key role in the activation or inhibition of the NLRP3 inflammasome. The quercetin also has an effect on these factors, thereby suppressing NLRP3 inflammasome and eventually inflammation. TH17 and its associated cytokines play an important role in inflammatory diseases through neutrophil recruitment. The neutrophil recruitment and activation in COVID-19 have also been well demonstrated. The inhibitory effects of quercetin on IL-17 can double the hope for a positive outcome in the treatment of COVID-19. Gut microbiota has an unparalleled function in the regulation of immune responses and the development of a variety of diseases caused by aberrant immune responses. The effect of quercetin on the correct dysbiosis can help control systemic inflammation in the body. Finally, the quercetin, as an anti-inflammatory, antioxidant, analgesic and NLRP3 inflammasome inhibitor compound, can be a potential treatment for severe inflammation, which is the main life-threatening condition in patients with COVID-19.

## Data Availability

The datasets used and/or analyzed during the current study are available from the corresponding author on reasonable request.
